# Compensatory reserve index during central hypovolemia and volume loading in healthy young and older hyperthermic adults: A pilot study

**DOI:** 10.14814/phy2.16177

**Published:** 2024-08-06

**Authors:** Josh Foster, Daniel Gagnon, Caitlin P. Jarrard, Whitley C. Atkins, Zachary McKenna, Steven A. Romero, Craig G. Crandall

**Affiliations:** ^1^ Institute for Exercise and Environmental Medicine Texas Health Presbyterian Hospital Dallas, University of Texas Southwestern Medical Center Dallas Texas USA; ^2^ Centre for Human and Applied Physiological Sciences, Faculty of Life Sciences and Medicine King's College London London UK; ^3^ Montreal Heart Institute and School of Kinesiology and Exercise Science Université de Montréal Montreal Quebec Canada; ^4^ Department of Physiology and Anatomy University of North Texas Health Science Center Fort Worth Texas USA

**Keywords:** age, compensatory reserve, heat, hyperthermia

## Abstract

The compensatory reserve index (CRI), derived from machine learning algorithms from peripherally obtained photoplethysmography signals, provides a non‐invasive assessment of cardiovascular stability, that may be useful clinically. Briefly, the CRI device provides a value between 0 and 1, with 1 reflecting full compensable capabilities and 0 reflecting little to no compensable capabilities. However, the CRI algorithm was developed in younger to middle aged adults, such that it is unknown if older age modulates CRI responses to cardiovascular challenges. In young and older subjects, we compared CRI responses to normothermic and hyperthermic progressive lower body negative pressure (LBNP), and volume loading with saline infusion. Eleven younger (20–36 years) and 10 older (61–75 years) healthy participants underwent (1) graded normothermic LBNP up to 30 mmHg, (2) graded hyperthermic (1.5°C increase in blood temperature) LBNP up to 30 mmHg, and (3) infusion of 15 mL/kg saline (volume loading) with hyperthermia maintained. CRI was obtained throughout each procedure. CRI at 30 mmHg LBNP was 0.18 and 0.24 units greater in the older group during normothermic and hyperthermic LBNP, respectively. However, CRI was not different between age groups at any other LBNP stage, nor did CRI change with volume loading regardless of age. In response to passive hyperthermia alone, regression analyses showed that heart rate was the strongest predictor of CRI. Blood temperature, rate pressure product, and stroke volume were also predictive of CRI but to a lesser extent. In conclusion, age attenuates the reduction in CRI during progressive normothermic and hyperthermic LBNP, but only at 30 mmHg. Second, the CRI was unchanged during volume loading in all subjects. Future studies should determine whether the age differences in CRI reflect age differences in LBNP tolerance.

## 1 INTRODUCTION

Hemorrhage is the leading cause of battlefield and civilian trauma deaths (Bellamy, 1984; Hoyt et al., 2008; Sauaia et al., 1995). Acute reductions in central blood volume initiates a compensatory response aimed at preserving arterial blood pressure and blood flow to vital organs. In the early stages of a hemorrhagic injury, these compensatory mechanisms include: (1) inhibition of the parasympathetic activity to increase heart rate, (2) activation of the sympathetic nervous system to increase peripheral vascular resistance, heart rate, cardiac contractility, and cardiac output, (3) the increased secretion of hormonal mediators to promote vasoconstriction, increased systemic vascular resistance, and preservation of intravascular fluid volume, and (4) sympathetic stimulation of venous capacitance vessels to increase venous return (Alexander, 1979; Evans et al., 2001; Mellander & Lewis, 1963; Schadt & Ludbrook, 1991). As the severity of hemorrhage progresses, the ability for these compensatory responses to preserve blood pressure decreases, leading to cardiovascular decompensation. The individual then transitions into a state of non‐compensable hemorrhage, manifested by profound parasympathetic stimulation (decreased heart rate) coupled with the withdrawal of sympathetic stimulation (thereby causing massive vasodilation), resulting in decreases in systemic vascular resistance and cardiac output (Barcroft & Edholm, 1945; Cooke et al., 2004; Evans et al., 2001; Sander‐Jensen et al., 1988; Schadt & Ludbrook, 1991). Arterial pressure then falls to the point where it is insufficient to perfuse vital organs, culminating in death.

The central hypovolemia associated with hemorrhage can be simulated experimentally using lower‐body negative pressure (LBNP), which draws blood away from the central circulation towards the capacitance vessels of the lower body (Crystal & Salem, 2015). Importantly, LBNP initiates cardiovascular responses similar to actual hemorrhage (Johnson et al., 2014; Rickards et al., 2015). Tolerance to graded LBNP decreases in heat stressed individuals (Borgman et al., 2019; Pearson et al., 2017) due to multiple mechanisms (Schlader et al., 2016), foremost is a heat‐induced redirection of blood from the central circulation to the skin reducing central blood volume prior to LBNP (Rowell et al., 1969). Subsequent hemorrhage (or graded LBNP) further decreases central blood volume, resulting in inadequate venous return and thus insufficient cardiac output to maintain organ perfusion pressure (Bundgaard‐Nielsen et al., 2010). Second, reductions in total body water due to profuse sweating in heated individuals can compromise LBNP tolerance by decreasing plasma volume and further contributing to central hypovolemia (Lucas et al., 2013).

The compensatory reserve index (CRI) was developed from machine learning algorithms that provide an estimate of how close an individual is to a state of hemodynamic decompensation (Convertino et al., 2020; Moulton et al., 2013). The device extracts a pulse oximetry waveform that is converted to a CRI value, from a proprietary algorithm, between 0 and 1, where 1 reflects a supine normovolemic state with full compensable capabilities, and 0 represents a state of little to no compensable capacity thereby approaching a condition of full cardiovascular decompensation (Moulton et al., 2013). The CRI is proposed to be used in several clinical situations (including remote settings such as in military combat) to determine two parameters. First, it can monitor the severity of hemorrhage and the progression of the patient while complementing other physiological indices such as heart rate and blood pressure (Convertino et al., 2020, 2021; Johnson et al., 2017, 2018). Second, it can be used to assess the effectiveness of treatments, such as volume loading with red blood cell transfusions in hemorrhaging patients (Schauer et al., 2021). Although CRI has been validated in the clinical setting in hemorrhaging patients, the independent effect of older age on CRI is unclear. The machine learning algorithm used to derive CRI from the pulse oximetry waveform was developed from data obtained in adults aged 18–55 years (Moulton et al., 2013), such that its validity in both children and older people requires further investigation. Previous research in a pediatric population demonstrates that age can significantly affect CRI at rest (Rodriguez et al., 2022). Specifically, median baseline CRI values were lower in those aged 1–2 years (0.25) compared to those aged 14–17 years (0.60). In these populations, heart rate predicted 85% of the CRI value, which explained the lower value in young children (due to elevated heart rates). If CRI is linked to heart rate, the reduced ability of older people to increase heart rate during hyperthermia and hyperthermic LBNP (Gagnon et al., 2016) may impact CRI outcomes.

Differential responses in young versus older adults have clinical importance since CRI may be used to detect (i) ensuing hemodynamic decompensation and (ii) the effectiveness of volume loading, which is the standard treatment for hemorrhaging individuals. Furthermore, although hyperthermia accelerates hemodynamic decompensation during central hypovolemia (Keller et al., 2009), it is unknown whether the CRI tracks the reduction in cardiovascular reserve in these conditions. In addition to age, the impact of acute volume expansion on CRI during actual or simulated hemorrhage also requires further investigation. In actively bleeding patients, red blood cell transfusion increases CRI over a time‐course of 20–30 min (Benov et al., 2017; Schauer et al., 2021). However, the clinical/real‐world nature of these studies likely yields substantial individual variation in CRI responses, and the interaction between volume loading and age has not been investigated. Our group compared the cardiovascular responses of young and older people to graded LBNP up to 30 mmHg, with and without superimposed hyperthermia (Gagnon et al., 2016). We also examined the effect of acute volume expansion on cardiovascular responses in hyperthermic young and older adults (Gagnon et al., 2017). This manuscript documents and analyzes the previously unreported CRI responses from these experiments.

The aim of this analysis was to (1) determine age‐related differences in CRI responses to graded LBNP with and without hyperthermia, and (2) determine age‐related differences in CRI responses to acute volume expansion. We hypothesized that, (1) CRI would be lower in older individuals during normothermic and hyperthermic LBNP, and (2) while hyperthermic, CRI is improved during volume loading, the extent of which being similar between older and younger individuals.

## 2 METHODS

### 2.1 Participants

The data presented in this manuscript were collected from 11 young (6 males, 5 females) and 10 older (4 males, 6 females) adults during an experimental protocol addressing questions unrelated to the focus of this work, the results of which have been published previously (Gagnon et al., 2016, 2017). Participant characteristics are shown in Table 1.

**TABLE 1:** Participant characteristics and time to increase T_blood_ by 1.5°C. Data are mean and standard deviation, with range in parentheses. The *p* value was obtained from independent samples *t*‐tests.

	Young (6M/5F)		Older (4M/6F)		*p*‐Value
Age (years)	26 ± 5	(20–36)	69 ± 4	(61–75)	–
Height (cm)	165 ± 6	(158–177)	165 ± 8	(152–178)	0.633
Pre weight (kg)	63.8 ± 9.7	(45.6–81.3)	68 ± 8.2	(54.3–78.6)	0.120
Body surface area (m^−2^)	1.70 ± 0.13	(1.45–1.99)	1.75 ± 0.14	(1.60–1.96)	0.181
Urine specific gravity	1.016 ± 0.008	(1.002–1.028)	1.018 ± 0.007	(1.005–1.028)	0.724
Heating time (min)	71 ± 12	(56–93)	78 ± 12	(67–109)	0.258
Sweat loss (L) before saline infusion^a^	1.18 ± 0.65	(2.31–0.47)	0.91 ± 0.69	(2.40–0.18)	0.386

^a^ Sweat loss before saline was calculated from nude pre to post body mass and correcting for volume of saline infused in the latter part of the protocol.

The participants were non‐smokers, free of known cardiovascular, respiratory, neurological, or metabolic diseases and none were taking prescription medications for the treatment of such diseases. Participants volunteered for one study visit that was performed at ~9 a.m. Participants were asked to refrain from strenuous physical activity for 24 h, as well as from caffeine and alcohol 12 h prior to the study visit.

### 2.2 Measurements

Urine specific gravity was assessed from a urine sample provided by the participants upon arrival to the laboratory using a handheld refractometer. If participants scored between 1.025 and 1.029, they were provided 500 mL water and the test continued. If any scores reached 1.030, the test was rescheduled to a later date. Continuous blood pressure measurements were obtained noninvasively (Finometer Pro, FMS, Amsterdam, the Netherlands). Continuous photoplethysmograph waveforms were obtained from a finger pulse oximeter (Nonin Medical Inc, Plymouth, MN), integrated with the CipherOx CRI system (V2.0.1, Flashback Technologies Inc, Boulder, CO), from which the CRI values were derived using proprietary algorithms. While proprietary, the developers suggest that ~200 features are analyzed from each individual waveform, reflecting the integration of all relevant compensatory mechanisms (Convertino & Sawka, 1985). This novel approach assesses the continuous pulsatile waveforms to estimate the patient's remaining reserve (from 0 to 1) prior to cardiovascular decompensation.

A 6F balloon‐tipped fluid‐filled catheter (Swan‐Ganz; Edwards Life Sciences) was placed under fluoroscopic guidance through an antecubital vein into a branch of the pulmonary artery. The catheter allowed for the measurement of pulmonary capillary wedge pressure and pulmonary artery blood temperature (T_blood_). More details on the measurement are available in our companion manuscripts (Gagnon et al., 2016, 2017). Forearm blood flow was measured using venous occlusion plethysmography (Whitney, 1953). Heart rate was obtained via a 5‐lead electrocardiogram that was interfaced with a cardiotachometer (CWE Inc.). Rate pressure product (an index of myocardial oxygen demand) was calculated as systolic pressur × heart rate (i.e., mmHg × bpm). Mean skin temperature (T_skin_) was measured as the weighted average of six thermocouples attached to the skin surface (Gagnon et al., 2015):

(1) $$\begin{array}{c} T_{\text{skin}}=0.22\text{chest}+0.21\text{upper back}+0.19\text{lower back}+\\ \;0.14\;\text{abdomen}+0.14\text{thigh}+0.11\text{calf} \end{array}$$

To increase T_blood_ and T_skin_, participants wore a tube lined, water perfused suit that covered the entire body except the head, hands, feet, and forearms. Only underwear (inclusive of sports bra for females) and shorts were worn under the suit. Stroke volume was determined by thermodilution from the pulmonary artery catheter (Gagnon et al., 2016, 2017).

### 2.3 Protocol

**FIGURE 1:**
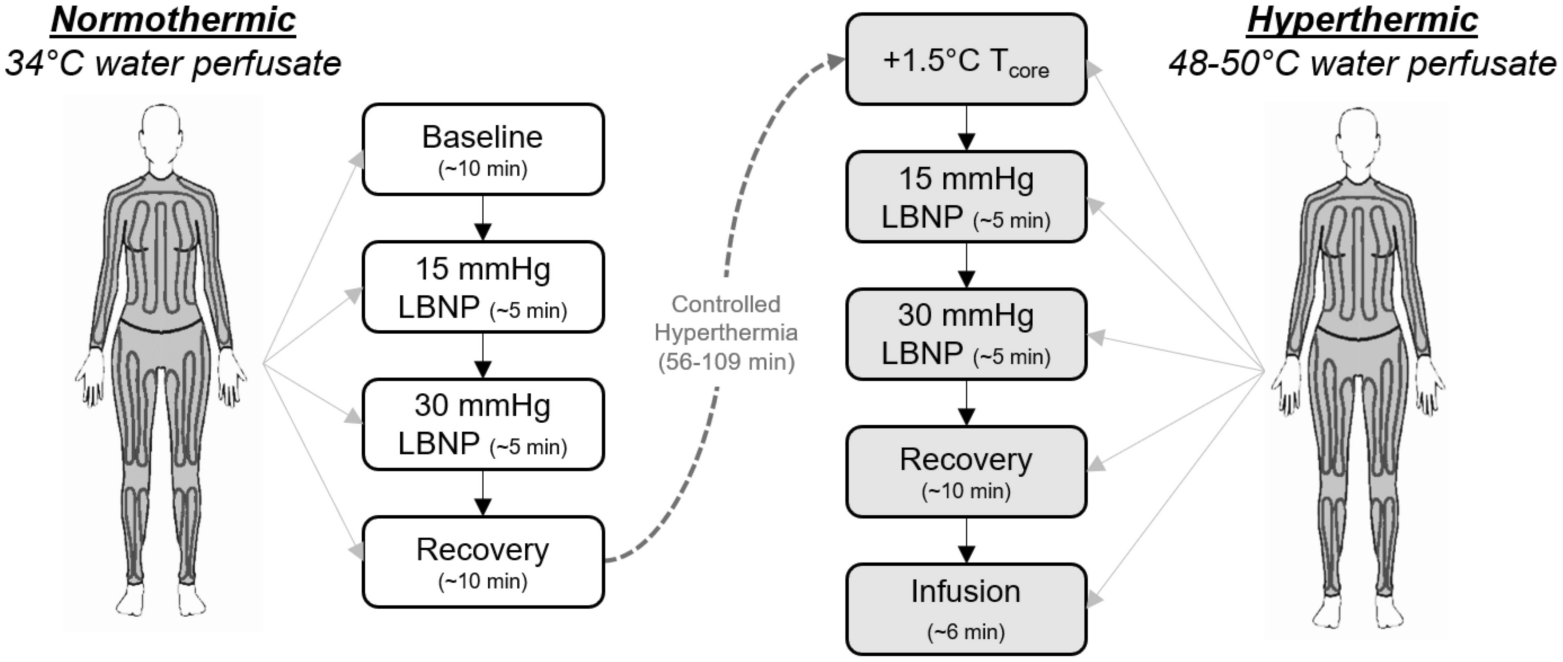
Study protocol. After being fitted with a tube lined suit perfused with 35°C water (suit shown right), participants were assessed during quiet rest/baseline (35°C water perfused through suit), and during progressive lower‐body negative pressure at 15 and 30 mmHg (LBNP, white boxes on the left). After recovery from 30 mmHg LBNP, pulmonary artery blood temperature (T_blood_) was increased by 1.5°C by perfusing 48–50°C water perfused through the suit. While hyperthermic, participants were then assessed during progressive LBNP at 15 and 30 mmHg. Finally, LBNP was ceased and participants rested and recovered while remaining hyperthermic. Assessments of CRI were then made in response to volume loading with body temperature controlled saline at a rate of 150–200 mL/min.

Participants volunteered for one study visit which was performed at approximately the same time of day for all participants (~9 am). To address the aims of our previously published studies (Gagnon et al., 2016, 2017), participants underwent right heart catheterization for the assessment of cardiac pressures, cardiac output by thermodilution, and T_blood_. Participants then lay in the supine position with their body sealed to the waist within a custom‐made LBNP device. The LBNP protocol was first initiated in a normothermic condition, with 34°C water perfused through the tube‐lined suit. Baseline measurements were made after 10 min of quiet rest. Thereafter, negative pressures of 15 mmHg (5 ± 1 min) and 30 mmHg (6 ± 2 min) were applied within the LBNP device. After 10 min recovery, baseline measurements were again obtained, followed by perfusing 50°C water through the tube‐lined suit to increase T_blood_. Once an increase in T_blood_ of 1.5°C had been attained, LBNP commenced at 15 mmHg (4 ± 1 min) and 30 mmHg (4 ± 1 min). The reason for choosing these levels of LBNP were not to replicate a specific level of blood loss per se. Instead, we wanted to compare LBNP responses between normothermia and hyperthermia, such that we needed to select an LBNP that would be less likely to cause pre‐syncope while in the heat (e.g., it is not uncommon for one to become presyncopal during 40 mmHg LBNP while heated). Upon cessation of LBNP, 15 mL/kg of warm saline, warmed to each individual's T_blood_, was rapidly infused (rate of 150–200 mL/min) through a separate intravenous catheter for 7 ± 1 min. Throughout this procedure, T_blood_ was maintained at 1.5°C above baseline by adjusting the temperature of the water perfusing the suit. After the infusion was completed, the individuals were cooled by perfusing cold water through the suit. A study protocol schematic is shown in Figure 1.

### 2.4 Statistical analysis

Statistical analyses were conducted in GraphPad Prism version 9. Data were checked for normality of distribution prior to group comparisons and passed in all instances. Data are therefore reported as mean ± standard deviation in data tables. Differences in subject characteristics between age groups were assessed with two‐tailed independent samples *t*‐tests. The mean difference and 95% confidence interval of the difference were used to describe age group differences. We used a two‐way mixed ANOVA to compare how CRI changed over LBNP stage (repeated factor) and between age groups (between factor). A Bonferroni posthoc correction was employed for multiple comparisons. For normothermic LBNP trials, three levels of LBNP stage were compared (baseline, 15 mmHg LBNP, and 30 mmHg LBNP) with two levels of age (young vs. old). For the hyperthermic LBNP trials, four levels of condition/LBNP stage were compared (pre‐heat baseline, +1.5°C T_blood_, 15 mmHg LBNP, and 30 mmHg LBNP) with two levels of age (young vs. old). To investigate the impact of volume loading (saline infusion) on CRI, two levels of time were compared (pre saline infusion vs post saline infusion), with two levels of age (young vs. old). The groups analysis were also completed for males and females separately (see Figure S1).

The *R* ^2^ value in linear regression represents the proportion of variation in CRI that is explained by the variation in a given variable. Thus, as an exploratory limb, linear regression was used to relate CRI values to the variables of interest, using one data point for each subject. The CRI data point used was from the hyperthermic data set (i.e., +1.5°C T_blood_) prior to LBNP. We only used one CRI data point per subject as to not violate the assumption of independence required for such analysis. Model intercepts and slopes between age groups were compared using the ‘are lines different’ tab in the model summary, where the software uses analysis of covariance (Zar, 1984). Pooled analyses including data for young and older participants were performed when analysis of covariance indicated that both the intercept and slope was not different between age groups (i.e., *p* > 0.05). As with our prior work (Watso et al., 2022) and based on current guidelines (Curran‐Everett, 2020), the alpha value for significance testing was set as *p* < 0.05 for the main outcome variables (i.e., CRI). Aside from CRI, *p* < 0.10 was considered to be statistically significant. In the context of simple linear regression, the *p* value reported represents the probability of a significant regression of Y on X.

## 3 RESULTS

### 3.1 Age differences in CRI response to normothermic LBNP

**FIGURE 2:**
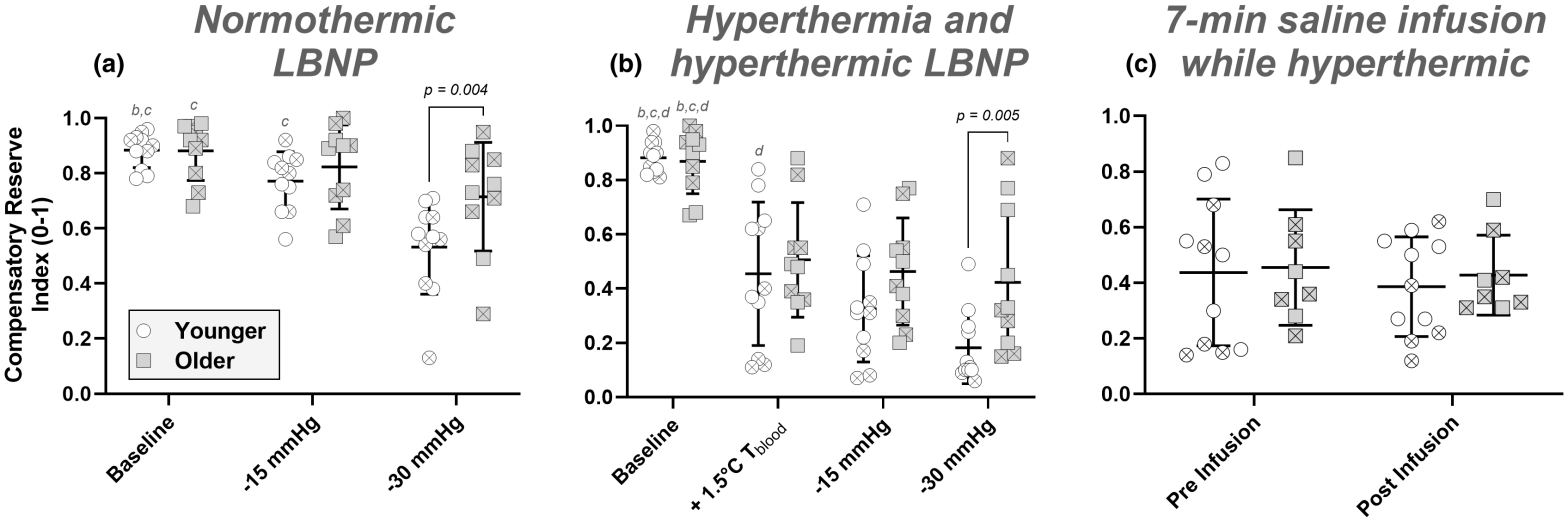
Compensatory reserve index to normothermic lower‐body negative pressure (LBNP) (a), hyperthermia and hyperthermic LBNP (b), and 6‐min volume loading with saline infusion (c). Between age group differences are shown with absolute *p* values. Letters correspond to *within subject* differences between LBNP stages, where *p* < 0.05. In graph a (normothermic LBNP), letters ‘b’, and ‘c’ indicate that stage is different from 15 mmHg LBNP, and 30 mmHg LBNP, respectively. In graph b (hyperthermia and hyperthermic LBNP), letters ‘b’, ‘c’ and ‘d’ indicate that stage is different from hyperthermia, 15 mmHg LBNP, and 30 mmHg LBNP, respectively. Young participants are shown with white circles, while older subejcts are shown with gray squares. Females are shown by hatched symbols.

The CRI responses are shown in Figure 2. There was no main effect for age group (*p* = 0.138). There was a main effect for LBNP stage (*p* < 0.001), and there was an interaction between age and LBNP stage (*p* = 0.011). In young adults, CRI was decreased at both 15 and 30 mmHg LBNP compared with baseline (*p* < 0.001 for each comparison). In contrast, older adults' CRI only decreased from baseline at 30 mmHg LBNP (*p* = 0.016). There was an age difference in CRI at 30 mmHg LBNP only, where it was 0.18 (95% CI: 0.03–0.33) greater in older participants (posthoc *p* = 0.012).

### 3.2 Age differences in CRI response to hyperthermia and hyperthermic LBNP

The CRI responses to hyperthermia and hyperthermic LBNP are shown in Figure 2. There was no main effect for age group (*p* = 0.136). There was a main effect for LBNP stage (*p* < 0.001), and there was an interaction between age and LBNP stage (*p* = 0.020). Similarly to normothermic LBNP, the only between age group difference was at 30 mmHg, where CRI was 0.24 (95% CI: 0.04–0.44) greater in older participants (post hoc *p* = 0.022).

### 3.3 Age differences in CRI response to saline infusion (volume loading) while hyperthermic

The CRI response to volume loading while hyperthermic is shown in Figure 2. There were no main effects for age group (*p* = 0.751), time (*p* = 0.225), and there was no interaction effect (*p* = 0.712).

### 3.4 Regression analysis for predicting CRI from thermoregulatory & cardiovascular data while individuals were hyperthermic before LBNP

#### 3.4.1 Age specific analyses

In young subjects, there was a relationship between CRI and heart rate (*R* ^2^ = 0.75, *p* < 0.001), T_blood_ (*R* ^2^ = 0.58, *p* = 0.006), stroke volume (*R* ^2^ = 0.54, *p* = 0.010), and RPP (*R* ^2^ = 0.65, *p* = 0.003). In older subjects, there was a relationship between CRI and heart rate (*R* ^2^ = 0.48, *p* = 0.026) and stroke volume (*R* ^2^ = 0.36, *p* = 0.067), although these relationships were weaker than those obtained from younger subjects.

In young or older subjects, there was no relationship between CRI and systolic (*R* ^2^ ≤ 0.10, *p* ≥ 0.341) and diastolic blood pressure (*R* ^2^ ≤ 0.01, *p* ≥ 0.836), pulmonary capillary wedge pressure (*R* ^2^ ≤ 0.04, *p* ≥ 0.581), or forearm vascular conductance (*R* ^2^ ≤ 0.05, *p* ≥ 0.468).

#### 3.4.2 Pooled analyses

Pooled analysis from combined data for both young and older participants demonstrates that CRI was related to heart rate (*R* ^2^ = 0.63, *p* < 0.001), RPP (*R* ^2^ = 0.45, *p* < 0.001), and T_blood_ (*R* ^2^ = 0.43, *p* = 0.002). The slopes (*p* ≥ 0.328) and intercepts (*p* ≥ 0.251) for each of these variables were not different between age groups.

Pooling data for young and older subjects, there was no relationship between CRI and systolic (*R* ^2^ = 0.01, *p* = 0.741) and diastolic blood pressure (*R* ^2^ = 0.00, *p* = 0.852), pulmonary capillary wedge pressure (*R* ^2^ = 0.00, *p* = 0.969), or forearm vascular conductance (*R* ^2^ = 0.04, *p* = 0.405).

## 4 DISCUSSION

The primary aim of this study was to examine whether the CRI is sensitive to age during central hypovolemia with and without hyperthermia. The secondary aim was to determine if the CRI is sensitive to volume loading while hyperthermic, given that volume loading improves tolerance to central hypovolemia and is therefore the main treatment method in hemorrhaging patients. In both groups, CRI was reduced during normothermic and hyperthermic LBNP, however CRI was higher in older individuals at 30 mmHg LBNP in both conditions (Figure 2). Second, while heat stressed, there was no change in CRI during ~7 min of volume loading (saline infusion) in both groups, such that CRI was not different between pre and post‐infusion regardless of age group. Finally, there was a strong linear relation between CRI and heart rate for the younger group, with the slope and intercept of the model not differing between age groups. This is the first study to demonstrate that age impacts the CRI response to central hypovolemia, with and without heat stress. Moreover, it is the first study to show that CRI does not increase with acute volume expansion during heat stress regardless of age, despite the known beneficial impact of volume loading on tolerance to central hypovolemia (Keller et al., 2009). Finally, we confirm prior investigations showing that absolute heart rate is a strong predictor of CRI (Rodriguez et al., 2022).

The CRI is a noninvasive method used to assess physiological reserve during central hypovolemia. If the CRI approaches zero, this indicates that an individual cannot make physiological adjustments to maintain blood pressure, suggesting imminent cardiovascular collapse. The CRI was developed through a machine learning algorithm, which analyzes how characteristics of the pulse oximetry waveforms change from full cardiovascular compensation to decompensation. The algorithm was developed in young healthy participants and accurately predicts tolerance to progressively increasing LBNP (Moulton et al., 2013), a model of hemorrhage. In clinical settings, CRI can be used in conjunction with traditional vital signs such as heart rate, blood pressure, end‐tidal carbon dioxide, and SPO_2_ to make treatment decisions (Convertino et al., 2020, 2021; Johnson et al., 2017, 2018). Our study, supported by previous research, confirms the sensitivity of CRI to central hypovolemia with LBNP (Moulton et al., 2013). The primary novel finding of this work is that we demonstrate an age‐related difference in CRI during normothermic and hyperthermic LBNP at 30 mmHg (Figure 2). At this level of LBNP, CRI was 0.18 and 0.24 units greater in the older group compared with the young group during normothermic and hyperthermic LBNP, respectively. Conversely, the reduction in CRI was not different between age groups: (a) during 15 mmHg LBNP (regardless of thermal condition), (b) in response to hyperthermia alone, and (c) in response to volume loading. Although heart rate was strongly related to CRI, heart rate alone only explained 63% of the variance in CRI, showing that additional factors not measured in the current study do factor into the CRI calculation obtained through machine learning (Moulton et al., 2013).

Although the CRI algorithm was not developed based on data from older people, it is likely that the observed heightened CRI in older people at 30 mmHg LBNP (while normothermic and hyperthermic) is physiologically valid and reflects greater compensatory reserve. In support of that statement, our companion paper shows that during both normothermic and hyperthermic LBNP, mean arterial pressure was greater in the older group compared with the younger group (Gagnon et al., 2016), implying greater “compensatory reserve”. Moreover, in a previous study comparing young (age ~ 26 years) and older (age ~ 64 years) males subjected to 50 mmHg LBNP, 5 of 13 (36%) younger males became acutely hypotensive (decrease in MAP by at least 15 mmHg), whereas only 1 of 13 (8%) of the older males became hypotensive (Taylor et al., 1992). Converse to those observations, older adults are more likely to become hypotensive initially during LBNP at 40 mmHg due to impaired chronotropic responsiveness (Shi et al., 2000). Overall, while the CRI's ability to predict tolerance requires validation in older participants (i.e., aged >55 years), the present findings demonstrate that CRI is indeed greater in older people during LBNP, at least during 30 mmHg.

Data from our lab and others show that volume loading improves LBNP tolerance in normothermic and hyperthermic individuals (Keller et al., 2009; Ligtenberg et al., 1998). Furthermore, our lab demonstrated that volume loading increases central venous and pulmonary capillary wedge pressures during LBNP (Gagnon et al., 2017), which likely explains increased tolerance. Although volume loading is used clinically to maintain hemodynamic stability during hemorrhage, the ability of the CRI to detect improvements in hemodynamic stability in a well‐controlled lab environment was previously unknown. Two previous studies investigated the impact of blood transfusion on CRI in real‐world hemorrhaging patients (Benov et al., 2017; Schauer et al., 2021). Those studies show that red blood cell transfusion increased CRI within 10–20 min following administration. However, the source of individual variation, and any potential effects of age on CRI responses to volume loading were not investigated. In contrast to the above cited studies, we saw no change in CRI following warmed saline infusion in hyperthermic young and older subjects. However, CRI was only monitored for 7 min during the infusion, which was a shorter duration relative to CRI changes in hemorrhaging patients (Benov et al., 2017). Moreover, it is unclear whether the physiological impact, inclusive of CRI responses, of red blood cell transfusion differs from saline infusion. It is also possible that saline infusion improves tolerance, as shown previously (Keller et al., 2009), through a mechanism not measured by the CRI device.

**FIGURE 3:**
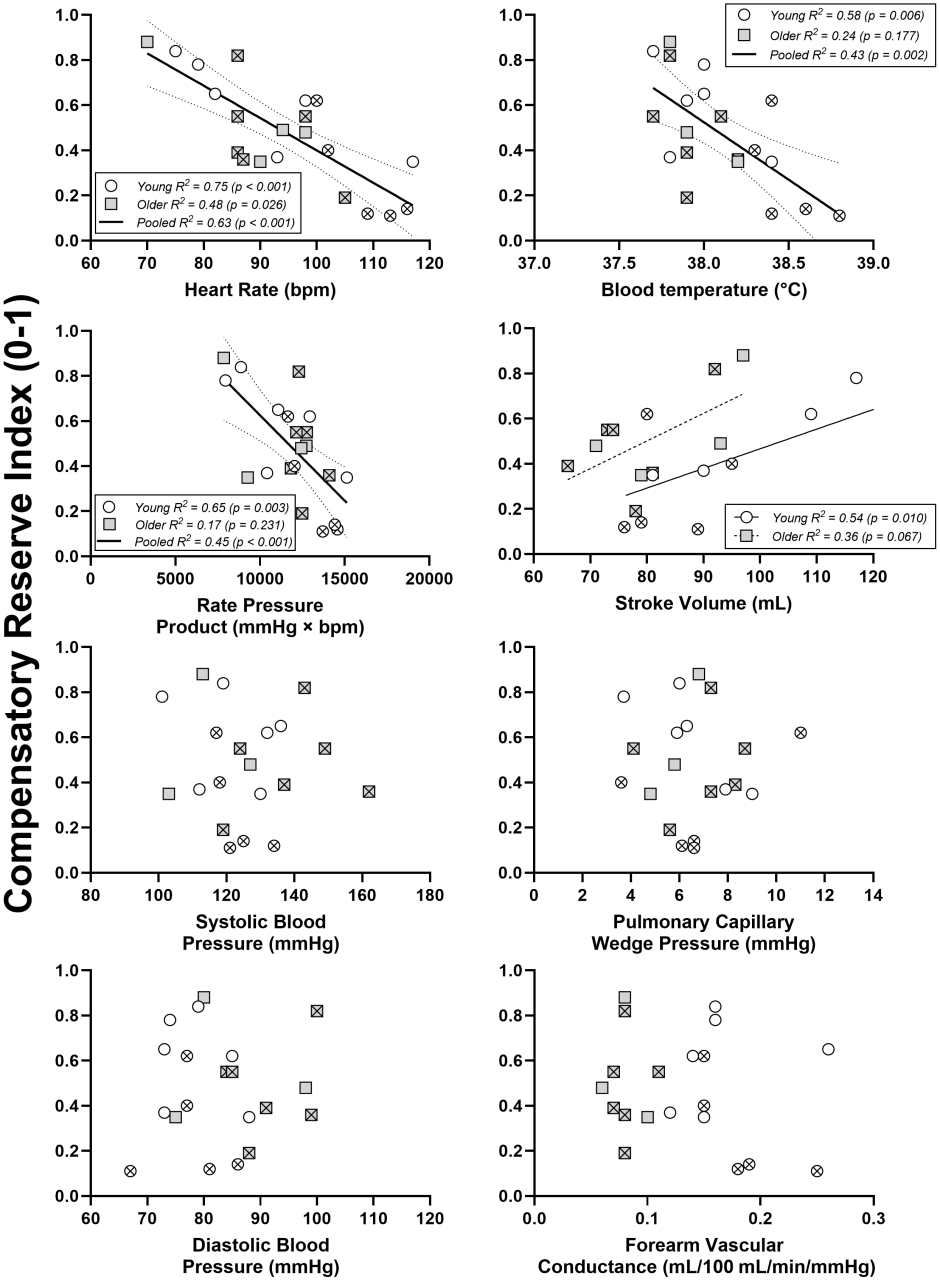
Relationship between compensatory reserve index and multiple variables. One data point per subject was used for this analysis. Data used are from the attainment of hyperthermia i.e., +1.5°C increase in pulmonary artery blood temperature with no lower body negative pressure. Where a pooled model adequately represented the data, this is shown by a single, bold line. Otherwise, dashed lines show the regression model for older subjects, and a solid line for younger subjects. Young participants are shown with white circles, while older subejcts are shown with gray squares. Females are shown by hatched symbols.

The present data show there is a negative relation between heart rate and CRI in young and older individuals (pooled *R* ^2^ = 0.63) such that CRI was greater when heart rate was lower. While prior work also shows a strong relation between CRI and heart rate in adolescents (Rodriguez et al., 2022), it is unknown if heart rate directly or indirectly impacts CRI since the algorithm is proprietary. However, heart rate may not have a consistently strong influence on CRI since there were no group differences in heart rate at normothermic 30 mmHg LBNP (Gagnon et al., 2016), yet differences in CRI were evident. Nonetheless, if heart rate directly impacts the calculation of CRI, this may explain why no changes in CRI were documented throughout volume loading, since heart rate is either unchanged or slightly elevated in response to volume loading in young and older individuals, respectively (Gagnon et al., 2017). While CRI was related to T_blood_ and stroke volume in both age groups (albeit to a weaker extent), CRI was not related to other cardiovascular variables such as systolic or diastolic blood pressure, pulmonary capillary wedge pressure, or forearm vascular conductance (Figure 3, *p* > 0.05).

Given the observed findings, future research directions include validation studies to determine if the observed age‐related differences in CRI during LBNP reflect changes in tolerance to progressive central hypovolemia. Such validation studies would confirm the CRI's utility in older individuals or provide a rationale for an update to the present algorithm used for its calculation. Second, while we included both males and females and different races/ethnicities in our cohort, we were not powered to detect differences in CRI between these populations. That said, prior work demonstrates that lower LBNP tolerance in normothermic females are consistent with lower compensatory reserve values (Ligtenberg et al., 1998; Schlotman et al., 2019). To investigate whether sex modified/skewed the interpretation of our results, we tested the CRI response to our protocol in male young vs older adults, and female young versus older adults specifically. While we were not powered a priori to test for sex differences, age differences in the CRI response to our protocol appear to be consistent between male and female subjects (Figure S1). Owing to the CRI's powerful utility for predicting cardiovascular decompensation, future work in general should seek to confirm the validity of the CRI in a variety of different populations (i.e., ethnic/race differences, chronic diseases) and in response to a wide variety of stressors (i.e., exercise, hypoxia, dehydration, etc).

### 4.1 Conclusions

In conclusion, the CRI value during normothermic and hyperthermic LBNP was greater in older individuals, but only at 30 mmHg. Second, the CRI was unchanged by volume loading of 15 mL/kg warmed saline in young or older hyperthermic adults. Third, heart rate was the strongest predictor of CRI in response to passive hyperthermia. To validate the heightened CRI values in older individuals at 30 mmHg LBNP, future studies should determine whether the age differences in CRI translate into differences in tolerance to progressive central hypovolemia.

## FUNDING INFORMATION

National Institutes of Health (NIH AG069005) and Department of Defense (W81XWH‐12‐1‐0152) to CGC.

## ETHICS STATEMENT

The study and informed consent documents were approved by the Institutional Review Boards at the University of Texas Southwestern Medical Center and at Texas Health Presbyterian Hospital Dallas (STU012014‐003). Written informed consent was provided by all participants prior to their participation in the study.

## Supporting information


Figure S1.
