# Supplementary material for: Compensatory reserve index during central hypovolemia and volume loading in healthy young and older hyperthermic adults: A pilot study
Source: Physiol Rep. 2024 Aug 6;12(15):e16177. doi: 10.14814/phy2.16177 (PMC11303067; doi:10.14814/phy2.16177)
Supplement: Supplementary file 1 — Figure S1. [file PHY2-12-e16177-s001.docx]

**Figure S1. Sex specific compensatory reserve index to normothermic lower-body negative pressure (LBNP) (a), hyperthermia and hyperthermic LBNP (b), and 6-minute volume loading with saline infusion (c).** Between age group differences are shown with absolute p values. Young participants are shown with white circles, while older subjects are shown with grey squares. Males are shown in the top panel, while females are shown in the bottom panel.
